# Purinergic Regulation of Endothelial Barrier Function

**DOI:** 10.3390/ijms22031207

**Published:** 2021-01-26

**Authors:** Muhammad Aslam, Dursun Gündüz, Christian Troidl, Jacqueline Heger, Christian W. Hamm, Rainer Schulz

**Affiliations:** 1 Experimental Cardiology, Department of Internal Medicine I, Justus Liebig University, Aulweg 129, 35392 Giessen, Germany; Dursun.Guenduez@med.uni-giessen.de (D.G.); christian.troidl@innere.med.uni-giessen.de (C.T.); christian.hamm@innere.med.uni-giessen.de (C.W.H.); 2 Department of Cardiology, Kerckhoff Clinic GmbH, 61231 Bad Nauheim, Germany; 3 DZHK (German Centre for Cardiovascular Research), Partner Site Rhein-Main, 61231 Bad Nauheim, Germany; 4 Department of Cardiology and Angiology, Evangelisches Jung Stilling Krankenhaus GmbH, 57074 Siegen, Germany; 5 Institute of Physiology, Justus Liebig University, 35392 Giessen, Germany; Jacqueline.Heger@physiologie.med.uni-giessen.de (J.H.); Rainer.Schulz@physiologie.med.uni-giessen.de (R.S.)

**Keywords:** Rac1, RhoA, peripheral actin, adenosine, ATP, ADP, UTP, endothelial permeability, oedema, P2X receptors, P2Y receptors

## Abstract

Increased vascular permeability is a hallmark of several cardiovascular anomalies, including ischaemia/reperfusion injury and inflammation. During both ischaemia/reperfusion and inflammation, massive amounts of various nucleotides, particularly adenosine 5′-triphosphate (ATP) and adenosine, are released that can induce a plethora of signalling pathways via activation of several purinergic receptors and may affect endothelial barrier properties. The nature of the effects on endothelial barrier function may depend on the prevalence and type of purinergic receptors activated in a particular tissue. In this review, we discuss the influence of the activation of various purinergic receptors and downstream signalling pathways on vascular permeability during pathological conditions.

## 1. Introduction

The vascular endothelium (VE), consisting of monolayers of endothelial cells (ECs), is located at the interface between the vascular and perivascular compartments and extends over a wide surface area. The VE separates strictly two compartments and regulates the trafficking of ions, solutes, macromolecules and leukocytes across the vessel wall, thus maintaining tissue homeostasis [1,2]. Additionally, it secretes several vasoactive agents that not only maintain its integrity but also regulate platelet function and vascular smooth muscle tone, and thus actively participate in the regulation of blood pressure. The semipermeable barrier function of VE is dependent on the size of the molecules, and this size-selective nature of the barrier to plasma proteins is a key factor in establishing protein gradients, which is required for fluid balance of tissues [1,3]. The loss of this barrier function of VE results in increased vascular permeability and leakage of blood components, which may finally result in organ dysfunction and life-threatening oedema formation [2,4].

Endothelial barrier integrity is maintained by the equilibrium of competing adhesive and contractile forces generated by adhesive molecules located at cell–cell and cell–matrix contacts and the acto-myosin-based contractile machinery, respectively [5]. ECs are tightly interconnected by the interaction of junctional proteins such as VE-cadherin, zona occluding 1 (ZO-1), occludins, and catenins that are linked to the actin cytoskeleton of adjacent cells [6,7]. Therefore, changes in the actin cytoskeleton dynamics and/or activation state of the EC contractile machinery may affect the stability of cell–cell junctions and barrier function.

Two members of the Rho family of GTPases, RhoA and Rac1, are the major regulators of endothelial actin cytoskeleton dynamics and contraction and thereby play a key role in the maintenance of endothelial barrier integrity. Constitutive activation of RhoA results in the loss of basal VE-cadherin and potentiates hypoxia-reoxygenation (H/R)-induced loss of endothelial barrier function, whereas suppression of RhoA activity attenuates the agonist-induced increase in endothelial permeability [8,9]. On the other hand, suppression of Rac1 activity in cultured ECs results in loss of the endothelial barrier and abolishes the recovery of EC barrier integrity following H/R-induced barrier failure. Accordingly, constitutive activation of Rac1 results in strong junctional staining of VE-cadherin and abrogates H/R-induced loss of cell–cell junctions [8].

**Figure 1:**
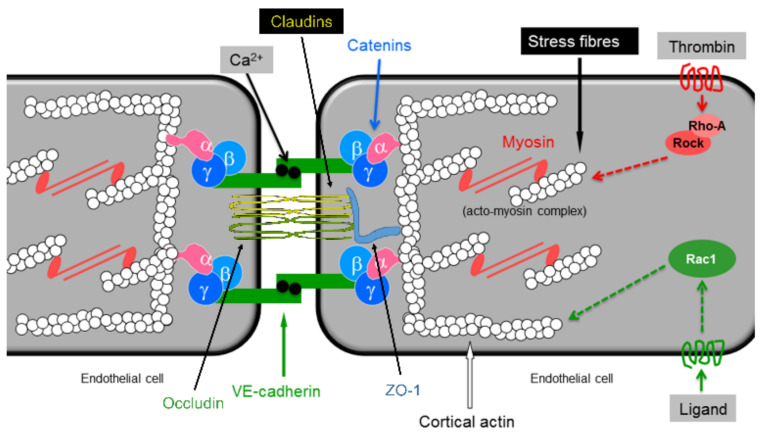
Schematic presentation of regulators of endothelial barrier properties. Rock: Rho associated kinase

The activation state of the endothelial contractile machinery is regulated by the phosphorylation state of regulatory myosin light chains (MLC), which are phosphorylated by MLC kinase (MLCK) [10] and dephosphorylated by MLC phosphatase (MLCP) [11]. Activation of Rho/Rho kinase (Rock) and MEK/ERK pathways induces MLC phosphorylation via inhibition of MLCP or activation of MLCK, respectively [12,13,14]. Thrombin inhibits MLCP by inducing the phosphorylation of its regulatory subunit MYPT1 at T850 and activates MLCK via phospholipase C/inositol tris-phosphate (PLC/IP3)-dependent release of Ca^2+^ from intracellular stores [12,14,15]. Both of these actions contribute to its endothelial barrier destabilisation properties. A schematic presentation of mechanisms regulating endothelial barrier properties is shown below (Figure 1).

Endothelial barrier integrity is influenced by several circulating, blood-borne hormones and agents/factors such as adenosine triphosphate (ATP) and its metabolites adenosine diphosphate (ADP) and adenosine. The major sources of vascular nucleotides are erythrocytes, platelets, and the endothelium [16]. Platelets contain nucleotides in their granules, and upon degranulation, bulk plasma levels of ATP can reach 50 μM [17], with even higher local concentrations predicted at the endothelial surface [18]. The endothelium releases nucleotides in response to shear stress [19], inflammatory mediators like thrombin [20], and hypoxia [21,22]. ATP and other nucleotides either released from vascular cells or applied exogenously can act at endothelial purinoceptors and modulate the barrier function of the endothelium [21,23,24]. Activation of purinergic receptors also induces the release of von Willebrand Factor (vWF) from ECs [25], which via reactive oxygen species (ROS)-dependent upregulation of endothelin-1 [26] may modulate endothelial barrier function.

## 2. Purine Receptors

There are two main classes of purine receptors: P1 receptors activated by adenosine and analogues, and P2 receptors recognised by purine and pyrimidine nucleotides (ATP, ADP, uridine triphosphate (UTP), uridine diphosphate (UDP)). P1 receptors are further divided into A_1_, A_2_, and A_3_ subtypes, depending on their affinity for adenosine. P2 receptors are further classified into ionotropic P2X and metabotropic P2Y receptors [27,28]. Nineteen different human purine receptors have been identified, cloned, and characterised [29]. Nearly all of these receptors are expressed on various cells of the cardiovascular system [27,29]. Several types of cells, particularly ECs and platelets, actively release nucleotides such as ATP that can activate a variety of the purine receptors in the vicinity [30,31]. This receptor activation scheme may be further complicated by the activity of ectonucleotidases that hydrolyse ATP to adenosine, which can activate P1 receptors [32].

## 3. Adenosine and Adenosine (P1) Receptors

Physiological extracellular adenosine levels range from 20 to 300 nM, which rise to a low micromolar range during exercise and to a high micromolar level under pathological conditions like ischaemia [33,34]. Under physiological conditions, the major source of extracellular adenosine is intracellular adenosine released by nucleotide transporters; however, under stress conditions, it is generated from its precursors ATP, ADP, and adenosine monophosphate (AMP) by the combined activities of extracellular ectonucleotidases, CD73 and CD39 [35]. Extracellular adenosine mediates its effects via adenosine receptors. There are four well-characterised adenosine receptors, namely adenosine A_1_, A_2A_, A_2B_, and A_3_, which are classified as high (A_1_, A_2A_, A_3_) or low (A_2B_) affinity for binding their parent physiological agonist, adenosine [36]. All four adenosine receptors possess seven transmembrane domains and belong to the family of G-protein-coupled receptors (GPCR) [37]. The A_1_ and A_3_ receptors are coupled to G_q_ and/or G_i/o_, whereas A_2A_ and A_2B_ are coupled to G_s_ proteins. Activation of A_1_ and A_3_ receptors results in inhibition of adenylyl cyclase (AC) activity, leading to reduction in cyclic AMP (cAMP) production and suppression of downstream signalling [37,38]. Their activation also leads to PLC/IP_3_-dependent release of Ca^2+^ from the endoplasmic reticulum (ER), protein kinase C (PKC) activation, and nitric oxide (NO) production [39,40,41,42]. In cardiomyocytes and neurons, activation of A_1_ adenosine receptors stimulates the opening and blockade of K^+^ channels and P- and N-type Ca^2+^ channels, respectively [43,44]. Activation of both A_1_ and A_3_ receptors leads to PKC-dependent and independent mitogen-activated protein kinase (MAPK) activation [45]. Activation of both adenosine A_2A_ and A_2B_ receptors results in activation of AC, enhanced cAMP production, and activation of downstream signalling [36]. Adenosine receptors are widely distributed throughout the nervous, cardiovascular, respiratory, urogenital, gastrointestinal, and immune systems. All adenosine receptors are expressed on various cells of the cardiovascular system, including ECs [37,46].

### 3.1. Adenosine Receptors and the Endothelial Barrier

Adenosine is a non-selective agonist for all adenosine receptors and produces differential effects on endothelial permeability of various vascular beds depending on the type of receptors expressed.

#### 3.1.1. Adenosine Receptors and Lung Microvascular Permeability

In the lung vasculature, adenosine signalling has largely been shown to enhance endothelial barrier properties and ameliorate agonist-induced hyperpermeability. In a mouse model of acute lung injury, knockdown of CD39 or inhibition of CD73, the two sequential enzymes responsible for adenosine production, resulted in development of severe lung oedema in response to ventilation compared with wild-type littermates. These animals were rescued by the addition of exogenous apyrase, suggesting a protective role played by adenosine [47]. Both adenosine A_2A_ or A_2B_ receptors seem to mediate the protective effects of adenosine in the lung [48,49]. In an isolated rat lung perfusion model of ischaemia/reperfusion (IR), a selective A_2_-receptor agonist reduced the IR-induced increase in microvascular permeability [50]. Pharmacological activation of adenosine A_2A_ and A_2B_ receptors protected against hypoxia and lipopolysaccharide (LPS)-induced development of lung oedema [51,52], whereas deletion of adenosine A_2A_ or A_2B_ receptors in mice resulted in loss of adenosine-mediated preservation of the lung microvascular endothelial barrier [51,52]. These protective effects are mediated via augmented production of cAMP and downstream activation of Rac1 [53]. Likewise, we have previously shown that elevation of intracellular cAMP via adrenomedullin receptor activation protects against lung oedema [54]. On the other hand, too much adenosine also seems to be detrimental for the lung vasculature. Deletion of adenosine deaminase, an enzyme responsible for adenosine degradation, resulted in severe respiratory distress and lung inflammation in mice [55]. However, deletion of A_2B_ receptors in these mice did not rescue but worsened the conditions, which were accompanied by enhanced loss of pulmonary barrier function [56], suggesting a protective role of A_2B_ receptors. In contrast to murine lungs, in feline lungs, adenosine A_1_ receptor activation mediates IR- and LPS-induced pulmonary microvascular barrier disruption [57,58]: perfusion with A_1_ receptor antagonists xanthine amine congener (XAC)/8-cyclopentyl-1,3-dipropylxanthine (DPCPX) ameliorates IR-induced lung injury and oedema in these animals. These species differences are probably due to differential expression of adenosine receptors in murine and feline lungs. Like A_2_ receptor activation, pharmacological activation of adenosine A_3_ receptors with a selective agonist also protects against reperfusion-induced lung oedema. This protective effect is lost in A_3_ knockout mice in vivo [59]. However, the mechanism of this protective effect is still elusive.

#### 3.1.2. Adenosine Receptors and the Blood–Brain Barrier

The blood–brain barrier is a highly specialised structure formed by a very tight monolayer of microvascular ECs that are distinct from ECs of other vascular beds [60]. The brain ECs form tight junctions consisting of claudins, occludins, VE-cadherin, junctional adhesion molecules (JAMs), and zonula occludens (particularly ZO-1). Human and murine brain microvascular ECs express adenosine A_1_ and A_2A_ receptors [61,62,63]. Adenosine causes an elevation of central nervous system (CNS) barrier permeability. In an elegant study, Carman et al. demonstrated that a stable adenosine analogue 5’-*N*-ethylcarboxamidoadenosine (NECA) and selective A_1_ and A_2A_ receptor agonists increased blood–brain barrier permeability to low-molecular-weight dextran [63]. These adenosine effects were attenuated in mice lacking either A_1_ or A_2A_ receptors [63]. Similarly, mice lacking CD73 had low levels of extracellular adenosine and were protected against experimental autoimmune encephalomyelitis-induced development of brain oedema and leukocyte infiltration [64]. Accordingly, inhibition of endothelial A_2A_ receptors protected mice against thromboembolic stroke-induced development of cerebral oedema and leukocyte infiltration [65]. Likewise, regadenson, a selective A_2A_ receptor agonist used clinically as a coronary vasodilator for myocardial perfusion imaging, increased permeability of the human blood–brain barrier in vitro [66] and in that of the mouse in vivo [63]. It has recently been shown that certain viruses and bacteria exploit this reaction of the blood–brain barrier to adenosine to open the barrier for their entry into the brain by increasing local production of adenosine, which causes transient opening of the blood–brain barrier and allows their entry to the central nervous system (CNS) [67,68]. Several groups have also recently tried to exploit this property of adenosine receptor activation to transiently open the blood–brain barrier for the local delivery of drugs to the CNS [69,70,71,72,73,74].

#### 3.1.3. Adenosine Receptors and Coronary Microvascular Barrier

As in the blood–brain barrier, adenosine receptor activation in the coronary microvasculature results in loss of barrier integrity. A_2_ receptor activation increased permeability of rat coronary microvascular ECs in vitro [75]. Infusion of adenosine in pigs on a high-fat diet resulted in increased cardiac microvascular permeability in vivo [76]. Similarly, Di Napoli et al. showed that DPCPX abrogates reperfusion-induced coronary hyperpermeability [77]. However, the authors used DPCPX at a concentration that blocks all adenosine receptors, suggesting A_2_ receptors were also antagonised. In line with these reports, we have previously demonstrated that reperfusion caused the release of ATP from isolated rat coronary microvascular ECs that was degraded to adenosine. Inhibition of either ectonucleotidases or adenosine receptors abrogated endothelial barrier failure, whereas addition of apyrase and ectonucleotidases worsened reperfusion-induced endothelial barrier failure [21]. In a follow-up study, we demonstrated that adenosine induced an increase in rat mesentery microvascular permeability in situ and cardiac oedema in vivo. These adenosine effects were blocked by adenosine receptor antagonists. Furthermore, we showed that these effects were due to cAMP-mediated disruption of the microvascular endothelial cytoskeleton [78]. In a related study, we demonstrated that adenosine induced cAMP production (via adenosine A_2_ receptors) in coronary microvascular ECs [79] that caused an inhibition of RhoA and Rac1 signalling [80]. This is in contrast to macrovascular ECs, where cAMP production inhibited RhoA/Rock signalling while activating Rac1 GTPase [81,82]. Inhibition of both RhoA and Rac1 results in complete breakdown of the EC cytoskeleton and disruption of cell–cell junctions [78,80]. Activation of Rac1 rescued these cells from the loss of endothelial barrier integrity [80].

#### 3.1.4. Adenosine Receptors and the Macrovascular Endothelial Barrier

**Figure 2:**
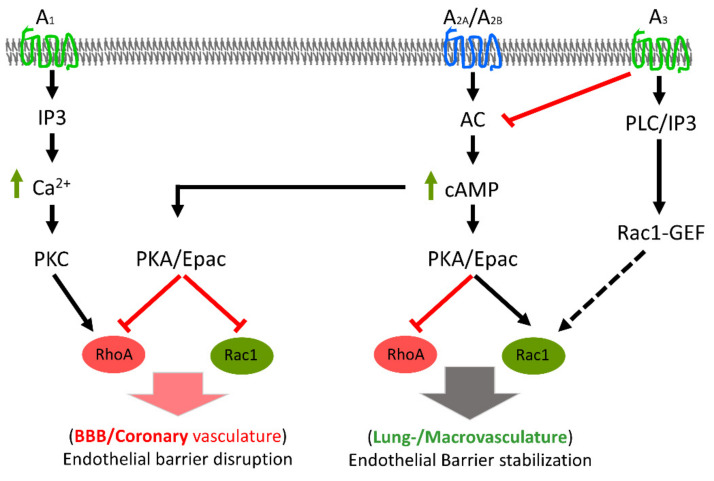
Key mechanisms involved in adenosine receptors-mediated endothelial barrier regulation. In lung microvasculature and macrovascular endothelium, A_2_ receptor activation causes an activation of Rac1 and an inhibition of RhoA, leading to stabilisation of the endothelial barrier. On the other hand, in coronary microvascular ECs, inhibition of both RhoA and Rac1 results in disruption of endothelial cytoskeleton and barrier failure. Black arrows indicate sequence of signal transduction, broken arrow indicates involvement of multiple steps in between, and green arrows indicate increase in cellular levels of indicated second messenger. Red bocks mean inhibition. AC: adenylyl cyclase; cAMP: cyclic adenosine monophosphate; GEF: guanine exchange factor; IP3: inositol triphosphate; PKA: protein kinase A; PKC: protein kinase C; PLC: phospholipase C.

In general, adenosine receptor activation in macrovascular ECs enhances endothelial barrier properties and ameliorates the effect of barrier-disrupting agents [79,83,84,85]. The mechanism involves the production of cAMP via activation of A_2A_ and A_2B_ receptors by adenosine and its analogues. Enhanced cellular cAMP levels suppress the activity of the endothelial contractile machinery in a RhoA/Rock-dependent manner and activate Rac1 GTPase via protein kinase A (PKA) and exchange protein directly activated by cAMP (Epac) activation [82]. Table 1 summarises the major preclinical studies that investigated purinergic receptors in relation to endothelial barrier function, and Figure 2 summarises the key mechanisms involved in the adenosine receptors-mediated endothelial barrier regulation in various vascular beds.

## 4. P2X Receptors and Signalling

The family of P2X receptors are non-selective ion channels comprising one or more of seven monomeric proteins (P2X1–P2X7). Each monomeric P2X protein consists of two transmembrane domains (TM1 and TM2) linked via an extracellular ligand-binding loop. The monomeric P2X proteins combine to form trimeric homomultimeric or heteromultimeric ion pores [96,97,98,99]; thus, each P2X receptor complex contains three ATP binding sites. At least 13 different trimeric combinations (P2X1, P2X2, P2X3, P2X4, P2X5, P2X7, P2X1/2, P2X1/4, P2X1/5, P2X2/3, P2X2/5, P2X2/6, and P2X4/6) have been reported and functionally characterised in vitro and partly in vivo [28,99]. Of note, P2X6 exists only in heteromeric combinations. Binding of ATP to the extracellular ligand-binding domain induces conformational changes in the multimeric ion pore, leading to opening of the pore and allowing the passage of ions into the cell. P2X receptors are generally known as non-selective cation channels, mainly permeable to Na^+^, K^+^, and Ca^2+^ under physiological conditions, although a recombinant P2X5 receptor has been shown to allow the passage of Cl¯. Excitable cells are thus depolarised upon activation of P2X receptors. Moreover, increased intracellular Ca^2+^ levels initiate a diverse array of Ca^2+^-dependent signalling pathways, both in excitable and non-excitable cells, that regulate various cellular processes, including cell migration, proliferation, necrosis, and apoptosis.

### P2X Receptors and Endothelial Barrier

**Figure 3:**
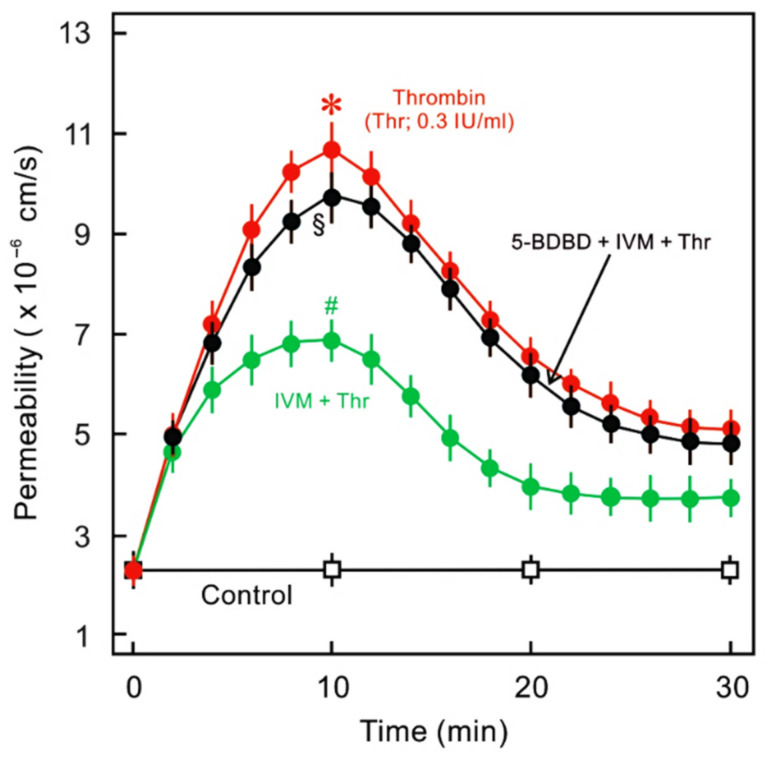
Effect of P2X4 receptor modulator (ivermectin; IVM) and antagonist ((5-(3-bromophenyl)-1,3-dihydro-2H-benzofuro[3,2-e]-1,4-diazepin-2-one: 5-BDBD) on thrombin-induced endothelial hyperpermeability. HUVEC monolayers cultured on filter membranes were exposed to human thrombin (Thr, 0.3 IU/mL) in the absence (red) or presence (green) of ivermectin (IVM; 50 µM) and the flux of labelled albumin was measured as described previously [84]. In a parallel set of experiments, P2X4 receptor antagonist (5-BDBD; 10 µM) was added before the addition of ivermectin and thrombin. *n* = 4, * *p* < 0.05 vs. control, # *p* < 0.05 vs. Thr alone, ^§^ *p* < 0.05 vs. IVM + Thr. For experimental details, please see methods in Supplementary File.

P2X receptors are widely expressed throughout the cardiovascular system. mRNA and protein of all P2X receptors have been detected in the endothelium of various types of blood vessels [100,101,102,103,104,105,106,107], but—with the possible exceptions of P2X4 and to some extent P2X7—their roles are unclear [100,103,108]. Human venous endothelium expresses higher levels of P2X4 than arterial endothelium [109]. The most studied human primary ECs are umbilical vein ECs (HUVECs), which express primarily P2X4 and P2X7 and low levels of P2X6 receptors [107] (unpublished data). P2X4 receptors mediate shear stress-induced Ca^2+^ currents in endothelium [110] that may be responsible for shear stress-mediated endothelial NO production and vasodilation [111]. The vessels from P2X4^(−/−)^ mice do not show an EC response to flow, such as calcium influx and subsequent production of NO [112]. A loss-of-function mutation in the human P2X4 receptor is associated with increased pulse pressure [113]. Cardiac ectopic expression of the P2X4 receptor was protective in a mouse model of heart failure [114]. Accordingly, the P2X4 receptor was the major regulator of ischemic preconditioning-mediated neuroprotection [87]. In HUVECs, the P2X4 receptor associates with VE-cadherin and may be involved in the regulation of cell–cell junctions [100]. In this context, we observed that ivermectin, a positive modulator of the P2X4 receptor, attenuated thrombin-induced HUVEC monolayer hyperpermeability (Figure 3). On the other hand, the P2X4 receptor is also reported to be an inflammation-regulated purinergic receptor. In rabbit aortic endothelium, the expression of P2X4 was upregulated after balloon injury followed by a high-fat diet [115]. A high-glucose and palmitate diet induced upregulation of P2X4 and P2X7 receptors accompanied by hyperpermeability of HUVEC monolayers that was attenuated by respective antagonists [116]. In line with this, ATP-mediated coronary microvascular endothelial barrier stabilisation was strengthened in the presence of P2X4 receptor antagonist (5-(3-bromophenyl)-1,3-dihydro-2H-benzofuro[3,2-e]-1,4-diazepin-2-one (5-BDBD)) and attenuated in the presence of the receptor modulator ivermectin [78]. Differential effects of P2X4 receptor activation on endothelial barrier function under different experimental conditions may be partly explained by the downstream signalling mechanisms. For example, under basal conditions, ECs express high levels of endothelial NO synthase (eNOS), which has been reported to be downregulated under chronic inflammatory conditions that may result in an upregulation of reactive oxygen species production, leading to barrier failure.

Unlike P2X4 receptors, activation of P2X7 receptors in ECs is primarily linked to a proinflammatory and hyperpermeability response. In an in vitro model of the blood–brain barrier, ATP induced an increased production of matrix metallopeptidase 9 (MMP9) in an interleukin (IL)-1β-dependent manner, which was responsible for the degradation of tight junction proteins [117]. These ATP effects were abrogated by P2X7 receptor antagonist, suggesting that they were P2X7 receptor-dependent. Similarly, hyperglycaemia induced the production of IL-1β via P2X7 receptor activation and caused damage to the retinal endothelial cell–cell junctions and barrier that was abrogated by a selective P2X7 receptor antagonist [118]. Likewise, in an in vivo model of intracranial haemorrhage, an upregulation of P2X7 receptor expression accompanied by the development of cranial oedema was observed. Pharmacological inhibition or siRNA-mediated knockdown of P2X7 receptors attenuated the disruption of the blood–brain barrier and the resultant oedema [88]. These effects were mediated via P2X7-induced activation of the RhoA/Rock pathway. Likewise, P2X7^(−/−)^ mice were protected against traumatic brain injury-induced development of brain oedema [89] and also the development of lung inflammation and oedema in vivo [119]. In contrast, Kaiser et al. [90] reported a protective role of P2X7 receptors in a cerebral transient IR model of brain injury and oedema formation. The mice deficient in P2X7 receptors developed more severe oedema after transient cerebral artery occlusion compared with their wild-type littermates [90].

## 5. P2Y Receptors and Signalling

P2Y receptors are membrane-bound class A GPCRs for extracellular nucleotides [120]. At present, eight mammalian P2Y receptor subtypes (P2Y_1_, P2Y_2_, P2Y_4_, P2Y_6_, P2Y_11_, P2Y_12_, P2Y_13_, and P2Y_14_) have been cloned and are further classified into two sub-families based on sequence similarities and signal transduction pathways [121,122,123,124,125,126,127,128]. The P2Y_1_-like subfamily includes the P2Y_1_, P2Y_2_, P2Y_4_, P2Y_6_, and P2Y_11_ receptors that are coupled to G_q_ proteins. The P2Y_11_ receptors are coupled additionally to G_s_ proteins, activation of which leads to an activation of AC and enhanced production of cAMP [128,129]. The P2Y_12_-like subfamily includes P2Y_12_, P2Y_13_, and P2Y_14_ receptors, which mediate cellular signalling via G_i_ proteins [128], activation of which leads to inhibition of AC and reduction in cellular cAMP levels [127,128]. Moreover, activation of several P2Y receptors is associated with activation of the MAPK pathway, and consequently these receptors are involved in cell survival and proliferation [123,130].

### P2Y Receptors and Endothelial Barrier

**Figure 4:**
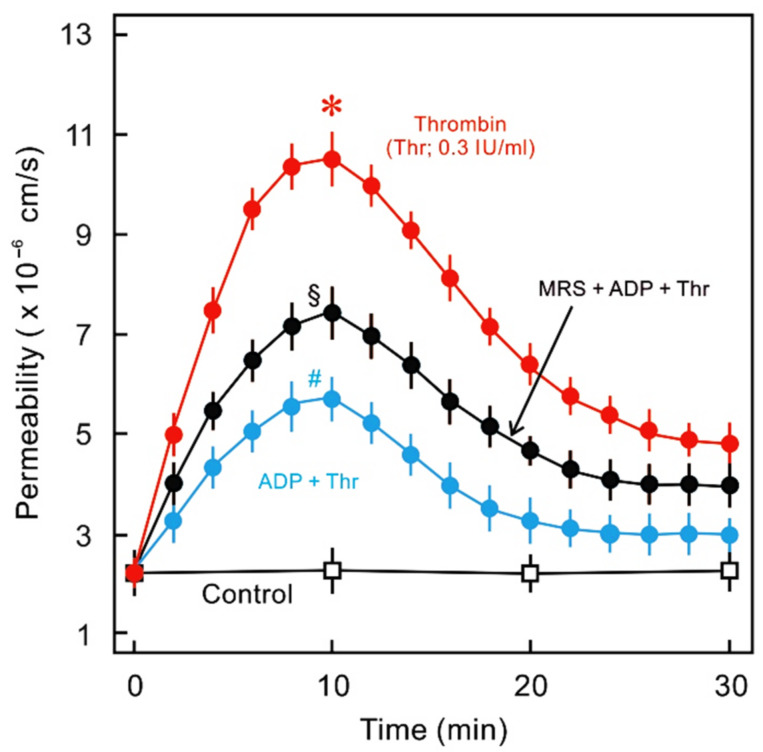
Effect of ADP and P2Y_1_ antagonist (MRS2500) on thrombin-induced endothelial hyperpermeability: HUVEC monolayers cultured on filter membranes were exposed to human thrombin (Thr, 0.3 IU/mL) in the absence (red) or presence (blue) of P2Y_1_ receptor agonist ADP (10 µM), and the flux of labelled albumin was measured as described previously [84]. In a parallel set of experiments P2Y_1_ receptor antagonist (MRS2500; 10 µM; black) was added before the addition of ADP and thrombin. *n* = 4, * *p* < 0.05 vs. control, # *p* < 0.05 vs. Thr alone, ^§^ *p* < 0.05 vs. ADP + Thr.

ECs express several of the P2Y receptor subtypes that are distributed over the entire vasculature. The endothelial P2Y receptors have been investigated primarily within the context of their NO-mediated vasorelaxant properties; therefore, fewer data are available in relation to their role in maintaining the endothelial barrier. The P2Y_1_ receptor is a ubiquitously expressed endothelial purinergic receptor on most EC types. It is a G_q_-linked GPCR that has been well-studied in platelet biology, for which ADP acts as a natural agonist and ATP an antagonist [131,132]. P2Y_1_ knockout mice are viable, fertile, normal in size, and do not present gross physical or behavioural abnormalities [133]. P2Y_1_^(−/−)^ homozygous mice are more susceptible to lung infections and are resistant to ADP/collagen-induced thrombin formation [133,134]. Moreover, P2Y_1_^(−/−)^ apoE^(−/−)^ double knockout mice have reduced amounts of atherosclerotic lesions [91] that were not affected by transplanting wild-type bone marrow to the knockouts, suggesting the vascular but not the haematopoietic P2Y_1_ receptor may be involved in the atherogenic response [91]. Moreover, leukocyte recruitment to inflamed vessels was reduced in vivo and leukocyte transendothelial migration was reduced in P2Y_1_ knockout as well as P2Y_1_ receptor antagonist-treated ECs in vitro [135]. These studies suggest that the P2Y_1_ receptor may potentiate vascular inflammation and hyperpermeability. However, in a mouse model of traumatic brain injury, development of cerebral oedema was ameliorated in mice treated with the P2Y_1_ agonist 2-methylthioadenosine 5′diphosphate (2MeSADP). These protective effects of the P2Y_1_ agonist were lost in inositol 3-phosphate receptor 2 (IP3R2)-knockout mice, suggesting that it is an IP3/Ca^2+^-dependent phenomenon [92]. We observed that P2Y_1_ mRNA is expressed in HUVECs, and treatment of cultured HUVEC monolayers with ADP as well as P2Y_1_-selective agonist 2MeSADP antagonised thrombin-induced HUVEC hyperpermeability (Figure 4). This barrier-protective effect of P2Y_1_ agonist is probably mediated via G_q_/IP3/Ca^2+^-dependent activation of Rac1 [136].

P2Y_2_ and P2Y_4_ are G_q_/G_11_-coupled receptors that are activated by both UTP and ATP [128]. Global deletion of the P2Y_2_ gene reduces shear stress-induced vasodilation and hypertension [137]. However, P2Y_2_-knockout mice show reduced inflammatory cell infiltration into injured vessels [138], and endothelial-specific deletion of the P2Y_2_ receptor in apoE^(−/−)^ mice results in reduced inflammatory response and increased plaque stability [93], suggesting a pathological role of chronic P2Y_2_ receptor activation under inflammatory conditions. Accordingly, knockdown of P2Y_2_ receptors in HUVECs ameliorated LPS-induced transendothelial migration of activated neutrophils [139].

P2Y_4_-null mice are viable but display microcardia (small hearts), suggesting that the P2Y_4_ receptor plays a role in postnatal heart development [140]. Interestingly, cardiac ECs and not cardiomyocytes express the P2Y_4_ receptor, and loss of the P2Y_4_ receptor in cardiac ECs results in reduced growth and migratory capacity in vitro [140]. Surprisingly, P2Y_4_ knockout mice are protected from myocardial ischaemic injury, cardiac inflammation, and fibrosis in a left anterior descending (LAD) coronary artery ligation model [94]. Moreover, P2Y_4_-knockout mice are protected from an LPS-induced cardiac microvascular hyperpermeability response. These data suggest that although the endothelial P2Y_4_ receptor is required for normal development of the heart in mice, its activation may induce vascular hyperpermeability under pathological conditions.

P2Y_6_ is a G_q_-coupled receptor activated by UDP [128] that is expressed on aortic and cerebral ECs [141,142]. Global loss of P2Y_6_ receptors results in macrocardia (larger heart), and mice lacking the P2Y_6_ receptor show an amplified pathological cardiac hypertrophic response [143]. However, vascular deficiency of P2Y_6_ receptors results in reduced vascular inflammation and ameliorated neointima formation in an atherosclerosis mouse model [95,144]. In contrast, inhibition of cerebral P2Y_6_ receptors with a selective antagonist aggravates development of cerebral oedema in a mouse model of ischaemic brain injury [145].

P2Y_11_ is the only known human P2Y receptor coupled to G_s_ [124,128,129]. The murine orthologue of the P2Y_11_ receptor does not exist or at least has not yet been identified. Moreover, the lack of selective agonists and antagonists for this receptor as well as specific detection tools (antibodies) make functional investigations of the P2Y_11_ receptor difficult [146]. We did not detect P2Y_11_ mRNA in HUVECs, but other EC types were not investigated. Presumably, if it is expressed in some EC type, one would expect its activation would raise intracellular cAMP levels that can interact with multiple signalling pathways, e.g., Rac1-dependent actin cytoskeleton rearrangement and MLCP-mediated inactivation of the contractile machinery, thus modulating endothelial barrier properties.

**Figure 5:**
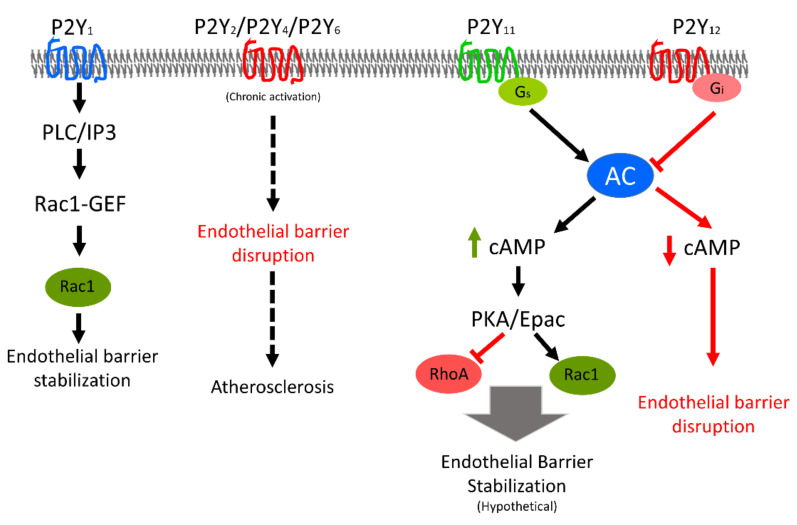
Schematic presentation of effect of various P2Y receptors’ activation on endothelial barrier function. Activation of P2Y_1_ receptor stabilises while chronic activation of P2Y_2_, P2Y_4_, and P2Y_6_ receptors results in atherosclerosis. Loss of endothelial barrier integrity is one of the early features of development of atherosclerotic plaques. The molecular mechanisms are not clear yet. P2Y_12_ receptor is G_i_-linked and its activation results in inhibition of adenylyl cyclase (AC) and reduction in intracellular cAMP content that leads to endothelial barrier destabilisation. The P2Y_11_ is G_s_-linked and its activation would lead to opposite effects and endothelial barrier stabilisation. The effects of P2Y_11_ are hypothetical based on available information about the P2Y_11_ receptor. Black solid arrows indicate sequence of signalling, broken arrows indicate multiple steps in between, green arrow shows increase in cellular cAMP levels, and red arrows indicate signalling via Gi leading to reduction in cAMP and endothelial barrier disruption. Red blocks mean inhibition.

The P2Y_12_-like subfamily comprises three members: P2Y_12_, P2Y_13_, and P2Y_14_. All of these receptors are coupled to G_i_, and their activation leads to suppression of AC activity and cAMP production [128]. P2Y_12_ is well-studied in relation to platelet biology, and its antagonists are used clinically as anticoagulants in various pathological conditions. In human cardiac-derived mesenchymal cells, ticagrelor, a P2Y_12_ receptor antagonist, induces the release of anti-apoptotic exosomes [147] that may also modulate the coronary microvascular endothelial barrier. Endothelial expression of both P2Y_12_ [84,148] and P2Y_13_ [104] has been documented. Recently, we demonstrated the expression of P2Y_12_ receptor mRNA and protein in HUVECs, and a specific P2Y_12_ antagonist increased intracellular cAMP levels and protected against thrombin-induced hyperpermeability [84]. We also observed the expression of P2Y_13_ but not P2Y_14_ mRNA in primary HUVECs (unpublished). In vasa vasorum ECs, ADP mediates a mitogenic response partly via P2Y_13_ receptors [104]. The expression of P2Y_14_ receptor has been reported in rat primary brain microvascular ECs [149], and activation of this receptor induces a pro-inflammatory response in ECs. Moreover, UDP-glucose (an agonist for P2Y_14_ receptor) mediated a contractile response in isolated pancreatic arteries in an endothelium-dependent manner, and this effect was abrogated by a selective P2Y_14_ receptor antagonist [150]. No further data are available related to the involvement of P2Y_13_ and P2Y_14_ receptors in the control of endothelial barrier properties. Figure 5 presents an overview about the effects of various P2Y receptors’ activation on endothelial barrier function.

## 6. Conclusions and Perspective

Endothelial barrier properties are influenced by extracellular nucleotides via activation of various purinergic receptors. The response depends on the type of receptor(s) present and the local concentration of the nucleotides. Adenosine, primarily via activation of A_2A_ and A_2B_ receptors, raises intracellular levels of cAMP in the lung microvascular bed and thus strengthens the barrier properties and ameliorates hypoxia- and inflammation-induced development of oedema. Selective agonists for adenosine A_2_ receptors are available that may be tested (for local application) for clinical use in various oedematous abnormalities of the lung, e.g., acute lung injury. Conversely, A_2_ receptor activation in brain and coronary microvasculature results in transient opening of the cell–cell junction in a cAMP-dependent manner. This property of the brain microvasculature can be exploited for local delivery of drugs to the CNS. P2 receptors are also widely distributed in the vascular bed. Chronic P2X receptor activation leads to endothelial barrier destabilisation and oedema formation, an effect primarily attributed to the P2X7 receptors. There is a need for the development of more selective and potent P2X7 receptor antagonists to ameliorate inflammation-induced loss of endothelial barrier function. There is also a lack of selective P2Y receptor agonists and antagonists, which makes the investigation of P2Y receptors in relation to endothelial barrier function difficult. We and others have documented that ATP at low micromolar concentrations stabilises endothelial barrier function, mainly via activation of various P2Y receptors, whereas at high concentration (in the millimolar range), it may act as a danger-associated molecular pattern (DAMP) [151], amplifying the inflammatory response. Inhibition of the P2Y_12_ receptor blocks inflammation-induced increases in endothelial permeability [84]. Further studies are needed to identify specific P2Y receptors that mediate endothelial barrier stabilisation and destabilisation.

## Figures and Tables

**Table 1:** Effect of purinergic receptor activation/inhibition on endothelial barrier of various vascular beds.

Receptor/Agonist	Model	Observation	Reference(s)
Adenosine	CD39 KO mice	Lung oedema	[47]
ATP + Apyrase	Rat heart perfusion in vivo	Increased oedema	[78]
A_1_ antagonist	Feline lung (IR) in vivo	Reduced lung oedema	[57,58]
A_1_ and A_2A_ KOsA_1_ and A_2A_ agonists	Mouse BBB in vivo	A_1_/A_2A_ agonists induced BBB permeability, effects lost in KOs	[63,66]
FDA approved A_2A_ agonist regadenoson	Rat model of brain drug deli-very	Increased BBB permeability of test drugs	[69,70,72,73]
A_2A_ agonist	Isolated pig lungs (IR)	Reduced lung oedema	[48]
A_2A_ KO/A_2A_ agonist	Lung permeability in vivo	A_2A_ agonist reduced lung permeability/Effect lost in A_2A_ KO	[51]
A_2B_ KO	Ventilator-induced lung injury	Increased lung oedema	[49]
A_3_ KO/A_3_ agonist perfusion	Lung IR (oedema) in vivo	A_3_ agonist reduced lung oedema/Effect lost in A_3_ KO	[86]
P2X4 antagonist	Brain middle artery occlusion (IPC-IR) mouse model	P2X4 antagonist abrogates protective effects of IPC	[87]
P2X7 antagonists	Rat intracranial haemorrhage/oedema	P2X7 antagonists alleviate oedema deve-lopment	[88]
P2X7 KO	Mouse traumatic brain injury	Reduced oedema development in KOs	[89]
P2X7 KO	Mouse middle cerebral artery occlusion	Aggravated oedema development in KOs	[90]
P2Y_1_/apoE double KO	Atherosclerosis	Reduced atherosclerotic plaques in double KOs	[91]
P2Y_1_ agonist	Mouse traumatic brain injury	P2Y_1_ agonist ameliorates oedema development	[92]
EC-specific P2Y_2_/apoE double KO	Atherosclerosis	Development of stable plaques in double KOs	[93]
P2Y_4_ KO	Myocardial infarction	Protection against myocardial infarction injury	[94]
P2Y_6_/apoE double KO	Atherosclerosis	Double KOs develop smaller and less inflamed lesions	[95]
P2Y_12_ antagonist	In vitro endothelial barrier model	P2Y_12_ antagonist ameliorates thrombin-induced hyperpermeability	[84]

ATP: adenosine 5′-triphosphate; BBB: Blood–brain barrier; EC: endothelial cell; FDA: United-States food and drug administration; IR: Ischaemia reperfusion; IPC: Ischaemic pre-conditioning; KO: Knockout.

## Data Availability

The data presented in this study are available within the manuscript.

## Supplementary Materials

The following are available online at https://www.mdpi.com/1422-0067/22/3/1207/s1.

## Abbreviations

AJ	Adherens junctions
ADP	Adenosine 5′-diphosphate
AMP	Adenosine 5′-monophosphate
ATP	Adenosine 5′-triphosphate
cAMP	3′, 5′-cyclic adenosine monophosphate
DAMP	Danger-associated molecular pattern
DPCPX	8-Cyclopentyl-1,3-dipropylxanthine
ER	Endoplasmic reticulum
ERK	Extracellular signal-regulated kinase
GPCR	G protein-coupled receptor
H/R	Hypoxia-reoxygenation
HUVEC	Human umbilical vein endothelial cells
I/R	Ischaemia reperfusion
LPS	Lipopolysaccharide
MAPK	Mitogen-activated protein kinase
MEK	MAPK/ERK kinase 1
MLCK	Myosin light-chain kinase
MLCP	Myosin light-chain phosphatase
MMP9	matrix metallopeptidase 9
MYPT1	Myosin phosphatase targeting subunit 1
NECA	5′-*N*-Ethylcarboxamidoadenosine
PKC	Protein kinase C
ROCK	Rho-associated kinase
ROS	Reactive oxygen species
UDP	Uridine diphosphate
UTP	Uridine triphosphate
XAC	Xanthine amine congener
VE	Vascular endothelium
vWF	von Willebrand Factor
ZO-1	Zonula occludens-1
