# Supplementary material for: Purinergic Regulation of Endothelial Barrier Function
Source: Int J Mol Sci. 2021 Jan 26;22(3):1207. doi: 10.3390/ijms22031207 (PMC7865261; doi:10.3390/ijms22031207)
Supplement: Supplementary file 1 [file ijms-22-01207-s001.pdf]

Materials and Methods

1. Materials.

ATP was from Roche (Mannheim, Germany); ThinCert® polycarbonate membrane filters (6-well) were from Greiner Bio-One (Frickenhausen, Germany); ADP and Human thrombin were from Sigma (Steinheim, Germany); MRS2500, 5BDBD, and ivermectin were from Tocris Bioscience (Bristol, UK);. All other chemicals were of the best available quality, usually analytical grade.

2. Cell culture.

The study conforms to the principles outlined in the ''Declaration of Helsinki'' (*Cardiovascular Research* 1997; 35: 2–3). HUVEC were isolated from umbilical cords obtained from gynaecology department of the University hospital Giessen after approval from the ethics committee of the hospital and informed consents from the patients. The cells were cultured as described previously [1] in complete EC culture medium (Cat # C-22010; PromoCell, Heidelberg, Germany) and used at passage 1-2.

3. Experimental protocols.

The basal medium used in incubations was modified Tyrode's solution (composition in mM: 150 NaCl, 2.7 KCl, 1.2 KH_2PO_4, 1.2 $MgSO_4$, 1.0 $CaCl_2$, and 30.0 *N*-2-hydroxyethylpiperazine-*N*'-2-ethanesulfonic acid; pH 7.4, 37° C). Agents were added as indicated. Stock solutions of ATP, ADP, MRS2500, and thrombin were prepared in water, 5BDBD and ivermectin in DMSO. Appropriate volumes of these solutions were added to the cells yielding final solvent concentrations ≤ 0.1% (vol/vol). Where combination of drugs was used, inhibitors were added 30-60 min before adding the agonist. The same final concentrations of water and DMSO were included in all respective control experiments.

4. Endothelial barrier properties.

The permeability of trypan-blue-labelled albumin across HUVEC monolayers was analysed as previously described [1, 2].

5. Statistical analysis.

The data are presented as means (± S.E.M) of 4 experiments from independent cell preparations. The comparison between multiple groups was performed by one-way analysis of variance (ANOVA) followed by a Student-Newman-Keuls post-hoc test using Graphpad Prism 6 software (Graphpad Inc.; San Diego CA, USA). The "P" values of ≤ 0.05 were considered statistically significant.

References:

1. Aslam, M.; Pfeil, U.; Gündüz, D.; Rafiq, A.; Kummer, W.; Piper, H.M.; Noll, T. Intermedin/adrenomedullin2 stabilises endothelial barrier and antagonises thrombin-induced barrier failure. *Br. J Pharmacol.* **2012**, 165, 208-222.
2. Gündüz, D.; Aslam, M.; Krieger, U.; Becker, L.; Grebe, M.; Arshad, M.; Sedding, D.G.; Härtel, F.V.; Abdallah, Y.; Piper, H.M.; Voss, R.K.; Noll, T. Opposing effects of ATP and adenosine on barrier function of rat coronary microvasculature. *J Mol.Cell Cardiol.* **2012**.
